# Clinical Research Progress in the Treatment and Prognosis of Comorbid Coronary Heart Disease and Lung Cancer

**DOI:** 10.31083/RCM48789

**Published:** 2026-06-15

**Authors:** Jiale Li, Yancai Kang, Fan Zhou, Junsheng Mu

**Affiliations:** ^1^Department of Cardiac Surgery, The Third Affiliated Hospital of Henan Medical University, 453000 Xinxiang, Henan, China; ^2^Department of Ultrasound, The Third Medical Center of People's Liberation Army of China General Hospital, 100039 Beijing, China; ^3^Department of Cardiac Surgery, Beijing Anzhen Hospital, Capital Medical University, Beijing Institute of Heart Lung and Blood Vessel Diseases, 100029 Beijing, China

**Keywords:** coronary artery disease, lung neoplasms, comorbidity treatment, prognosis analysis, clinical research progress

## Abstract

Driven by global population aging and increasingly unhealthy lifestyles, the number of patients with comorbid coronary heart disease (CHD) and lung cancer is increasing, as these two diseases share common risk factors, such as smoking. There is a core contradiction in the clinical management of these diseases: radical treatment for lung cancer may increase the cardiac burden, whereas the long-term antithrombotic therapy required for CHD conflicts with the risk of bleeding during lung cancer surgery. This review summarizes recent advances in the treatment and prognosis of these comorbid diseases based on literature from the past decade. Individualized treatment is necessary: for patients with early-stage lung cancer, limited coronary artery disease, and good cardiac function, synchronous surgery may be considered; conversely, for patients with advanced lung cancer, complex coronary artery disease, or cardiac dysfunction, staged surgery is more secure, and minimally invasive techniques can help reduce trauma and accelerate recovery. Non-surgical treatments require adjustment of the radiation dose and the use of prophylactic antithrombotic therapy to balance efficacy and cardiac protection. Prognostic evaluations have shown that a low inflammatory index, extensive coronary calcification, a high SYNTAX score, and comorbid diabetes mellitus are independent risk factors for poor outcomes in lung cancer patients with CHD.

## 1. Introduction

### 1.1 Research Background

Under the effects of global population aging and the prevalence of unhealthy lifestyles, such as smoking and obesity, coronary heart disease (CHD) and lung cancer have become the major diseases. Because these two diseases are more prevalent in middle-aged and elderly people and share common risk factors, such as smoking and hypertension, the number of patients with comorbid CHD and lung cancer is increasing [1,2]. Clinical data indicate that the comorbidity rate of CHD with lung cancer among elderly patients ranges from 5.3% to 24%. However, it should be noted that these data are based on pooled results from multiregional clinical studies covering populations in parts of Asia and Europe, without being limited to a single region; therefore, due to the current lack of globally unified epidemiological survey data, these findings cannot be definitively identified as overall global data. The treatment of comorbid CHD and lung cancer faces dual challenges: radical treatments for lung cancer (e.g., surgery, radiotherapy) may increase the cardiac burden and induce cardiovascular events, while revascularization therapies for CHD (e.g., percutaneous coronary intervention [PCI], coronary artery bypass grafting [CABG]) require long-term antithrombotic therapy, which conflicts with the risk of bleeding during lung cancer surgery. Thus, the selection of treatment strategies for comorbid CHD and lung cancer has become a crucial challenge in clinical practice.

### 1.2 Research Significance

Although a certain amount of data has been accumulated in current clinical research on CHD complicated with lung cancer, there remains a lack of unified standards in core areas, including treatment strategies (e.g., choice between simultaneous surgery or staged surgery, the role of PCI- as an alternative to surgical revascularization, and the integration of hybrid approaches combining PCI with thoracic surgery), prognostic factors (e.g., nutritional-inflammatory status, severity of coronary artery lesions), and innovations in diagnosis and treatment technologies (e.g., minimally invasive fusion techniques, simultaneous screening protocols). Furthermore, some research conclusions remain controversial. A systematic collation of research findings in this field, which can clarify the boundaries of safety and efficacy for different treatment regimens and identify key indicators influencing patient prognosis, can not only provide evidence-based findings for clinicians to formulate individualized treatment strategies, including the optimal use of medical therapy such as novel lipid-lowering agents and newer cardioprotective classes like glucagon-like peptide-1 receptor agonists (GLP-1 RAs) or dual GLP-1/glucose-dependent insulinotropic polypeptide (GIP) agonists in patients with comorbid CAD and lung cancer, but also reveal the gaps and deficiencies in existing research, indicate directions for future studies, and ultimately improve the quality of life and long-term survival rate of patients with these comorbid diseases.

### 1.3 Research Scope, Structure, and Literature Screening Methods

#### 1.3.1 Research Scope and Structure

This review is based on literature related to CHD complicated with lung cancer published from 2016 to 2025. It focuses on three core directions (optimization of treatment strategies, prognostic influencing factors, and innovations in diagnosis and treatment technologies) to systematically integrate the existing research findings. The structure of the review is as follows: first, it elaborates on the research status of treatment strategies for CHD complicated with lung cancer, comparing the efficacy differences between simultaneous surgery and staged surgery, as well as minimally invasive surgery and traditional surgery; second, it analyzes the key factors affecting patient prognosis, including nutritional-inflammatory indicators and characteristics of coronary artery lesions; third, it summarizes the innovative progress in comorbidity treatment technologies and indicates the current research controversies and future exploration directions, forming a logically complete research review system.

#### 1.3.2 Literature Screening Methods

Databases Searched: Comprehensive searches were conducted in core Chinese and English databases, including PubMed, Embase, Cochrane Library, China National Knowledge Infrastructure (CNKI), Wanfang Database, and VIP Chinese Science and Technology Periodical Database, to ensure the comprehensiveness of literature coverage.

Keyword Combinations: English keywords included “Coronary Artery Disease”, “Lung Neoplasms”, “Comorbidity”, “Synchronous Surgery”, “Staged Surgery”, “Prognosis”, “Minimally Invasive Treatment”, “Radiotherapy”, and “Antithrombotic Therapy”. A combined keyword search strategy was adopted to avoid missing relevant literature.

Inclusion Criteria: (1) patients with confirmed CHD complicated by lung cancer as study subjects. (2) research focused on treatment strategies (surgical/non-surgical), prognostic factors, or diagnostic/treatment innovations. (3) study types: randomized controlled trials (RCTs), cohort studies, case-control studies, systematic reviews, Meta-analyses. (4) publication date: January 2016–December 2025. (5) full-text articles with complete data and clear conclusions

Exclusion Criteria: (1) unclear CHD/lung cancer diagnostic criteria or incomplete baseline data. (2) low clinical evidence literature: case reports, review articles, animal or *in vitro* studies. (3) duplicate content or overlapping study populations. (4) unextractable key data or critical methodological flaws compromising result reliability.

## 2. Research Status of Treatment Strategies for Coronary Heart Disease Complicated With Lung Cancer

### 2.1 Surgical Treatment Strategies: Comparison Between Synchronous Surgery and Staged Surgery

Surgical treatment is the main intervention for resectable CHD complicated with lung cancer (Table 1). However, the choice of surgical timing (synchronous or staged) as well as the consideration of non-surgical coronary revascularization (PCI) and hybrid strategies has long been a focus of clinical controversy, and existing research has formed a relatively rich evidence chain [3].

**Table 1.** **Various surgical types**.

Surgical type	Core definition
Synchronous Surgery	A single surgical procedure that simultaneously completes cardiac coronary artery revascularization and lung cancer resection.
Staged Surgery	The cardiac surgery and lung cancer surgery are performed in two separate procedures.
Minimally Invasive Surgery	Surgical techniques with less trauma, such as small incisions or laparoscopy/thoracoscopy.
Hybrid Surgery	An integrated surgical approach combining interventional therapy and thoracoscopic surgery.

#### 2.1.1 Safety and Applicable Population for Synchronous Surgery

Synchronous surgery refers to the completion of coronary revascularization (e.g., CABG) and lung cancer resection in a single operation, and has the core advantages of avoiding the risk of tumor progression during staged surgery and reducing the trauma of secondary surgery for patients [4]. Multiple single-center retrospective studies have confirmed the feasibility of synchronous surgery in strictly selected patients. Li et al. [5] analyzed 20 patients who underwent synchronous CABG and lung cancer resection, showing that although the operation time (366.88 ± 94.48 min) and intraoperative blood loss in these patients were significantly higher than the corresponding values in patients who underwent lung cancer surgery only, there were no significant differences in the 5-year recurrence-free survival rate and 5-year overall survival rate. These findings suggest that synchronous surgery has comparable long-term efficacy to surgery alone. Lianyong et al. [6] explored minimally invasive synchronous surgery, completing minimally invasive CABG combined with thoracoscopic lung cancer resection through a left anterolateral small incision. In their study, all 16 patients achieved successful surgery with no serious complications, such as myocardial infarction or death, during the perioperative period, and the median ICU stay after surgery was only 60 h, indicating that minimally invasive techniques can further reduce the trauma and recovery time of synchronous surgery. Nevertheless, the applicable population for synchronous surgery remains strictly limited. Niu and colleagues [7] divided 84 patients into a simultaneous off-pump coronary artery bypass grafting group, a staged surgery group after PCI, and a non-revascularization group [8]. They found that synchronous surgery only showed advantages among patients with early-stage lung cancer (Stage I–II) and limited coronary artery lesions (single-vessel or double-vessel lesions); in patients with severe multivessel coronary artery lesions (SYNTAX score >15) or advanced lung cancer (Stage III and above), the incidence of coronary complications (28.6%) was significantly higher than that in the staged surgery group (12.3%), suggesting that synchronous surgery requires strict control of the patient selection criteria to avoid blind application in high-risk populations.

#### 2.1.2 Clinical Application and Risk Control of Staged Surgery

**Fig. 1.**
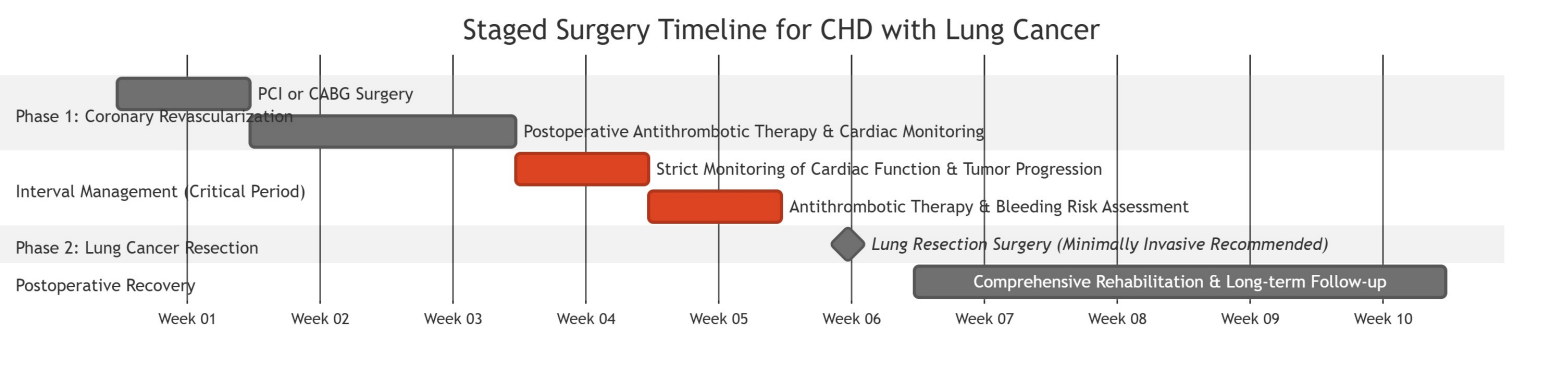
**Staged surgery timeline**.

Staged surgery usually adopts the timing of coronary revascularization first and lung cancer resection second, with the core goal of reducing the risk of perioperative cardiac events. However, it faces the contradiction between tumor progression and antithrombotic therapy. Yun et al. [9] showed that the incidence of perioperative cardiovascular complications in the staged surgery group (PCI first, lung cancer resection second) was significantly lower than that in the synchronous surgery group (9.5% vs. 18.2%), but the interval between the two operations must be strictly controlled. Specifically, if the interval exceeded 3 months, the progression rate of lung cancer T stage could reach 14.3% (2/14) (due to the small sample size, this result has certain limitations and needs to be verified by larger sample studies); if the interval was excessively short (<2 weeks), there was an increased risk of in-stent thrombosis after PCI (incidence rate, 3.1%). Therefore, they recommended that the interval between the two operations should be 2–3 weeks. The authors explicitly noted that due to the small sample size, the result regarding tumor progression rate has certain limitations and needs verification by larger sample studies. Reshetov et al. [10] conducted a long-term follow-up study on patients with severe CHD (requiring CABG) complicated with non-small cell lung cancer (NSCLC), comparing the prognosis of the staged surgery group (CABG first, lung cancer resection after an interval of 4–6 weeks) and the lung cancer surgery only group. Although this study provides valuable long-term survival data, its retrospective nature and potential selection bias indicate the need for prospective validation. They found that the 5-year postoperative survival rate in the staged surgery group was significantly higher than that in the pure surgery group (64.5% vs. 48.2%), with no significant difference in the recurrence-free survival rate (*p* = 0.90), suggesting that staged surgery may be a safer choice for patients with severe coronary artery lesions. However, attention should be paid to the interval risk in staged surgery. Andrushchuk et al. [11] reported that during the interval in staged surgery (average, 4.2 weeks), two patients died of acute coronary events, indicating that antithrombotic management and cardiac function monitoring should be strengthened during the interval to reduce the probability of adverse events (Fig. 1).

#### 2.1.3 Core Basis for the Selection of Surgical Strategies

Based on current research, the selection of surgical timing should be based on three dimensions: the severity of the coronary artery lesions, the lung cancer stage, and the overall condition of the patient. It is recommended to adopt the following clinical decision algorithm for systematic assessment and recommendation (Table 2)

**Table 2.** **Clinical decision algorithm for surgical strategy selection in patients with comorbid coronary heart disease and lung cancer**.

Assessment dimension	Low risk (Prioritize synchronous surgery)	High risk (Prioritize staged surgery)	Recommended surgical strategy
Lung Cancer Stage	Stage I–II	Stage III and above	Early-stage lung cancer may be considered for synchronous surgery; advanced lung cancer is recommended for staged surgery to prioritize tumor or cardiac risk control.
Coronary Artery Lesions	Single-/double-vessel disease; SYNTAX Score ≤15	Severe multi-vessel disease (especially SYNTAX Score >15)	SYNTAX Score >15 is an independent risk factor. Staged surgery or hybrid surgery is recommended for such patients to reduce perioperative cardiac complications.
Cardiac Function	LVEF >50%	LVEF ≤40%	LVEF ≤40% indicates cardiac dysfunction. Coronary revascularization (PCI/CABG) should be prioritized, and lung cancer surgery timing should be re-evaluated after cardiac function stabilizes.
Nutritional-Inflammatory Status	ALI >35 (indicating favorable status)	ALI <15 (indicating malnutrition and systemic inflammation)	ALI <15 is significantly associated with increased all-cause mortality. Preoperative nutritional support and anti-inflammatory intervention are warranted, with cautious assessment of surgical tolerance.
Other Risk Factors	No severe Diabetes Mellitus (DM); Low Coronary Artery Calcification (CAC) score	Comorbid Diabetes Mellitus (DM); CAC score ≥400 (especially when incidentally detected on low-dose CT screening)	Comorbid DM is an independent poor prognostic factor. A CAC score ≥400 increases the risk of 30-day postoperative cardiovascular complications by 3.2-fold, necessitating enhanced perioperative cardiac monitoring and risk management.

LVEF, left ventricular ejection fraction; PCI, percutaneous coronary intervention; CABG, coronary artery bypass grafting; ALI, advanced lung cancer inflammation index; CT, computed tomography.

For patients with Stage I–II lung cancer, single-vessel or double-vessel coronary artery lesions, and good cardiac function (left ventricular ejection fraction [LVEF] >50%), synchronous surgery (especially minimally invasive synchronous surgery) can be the preferred option; for patients with Stage III and above lung cancer, severe multivessel coronary artery lesions (SYNTAX score >15), or cardiac insufficiency (LVEF ≤40%), staged surgery (PCI/CABG first, lung cancer resection after an interval of 2–3 weeks) is more in line with safety principles [6,7].

### 2.2 Innovative Application of Minimally Invasive Techniques in Surgical Treatment

**Fig. 2.**
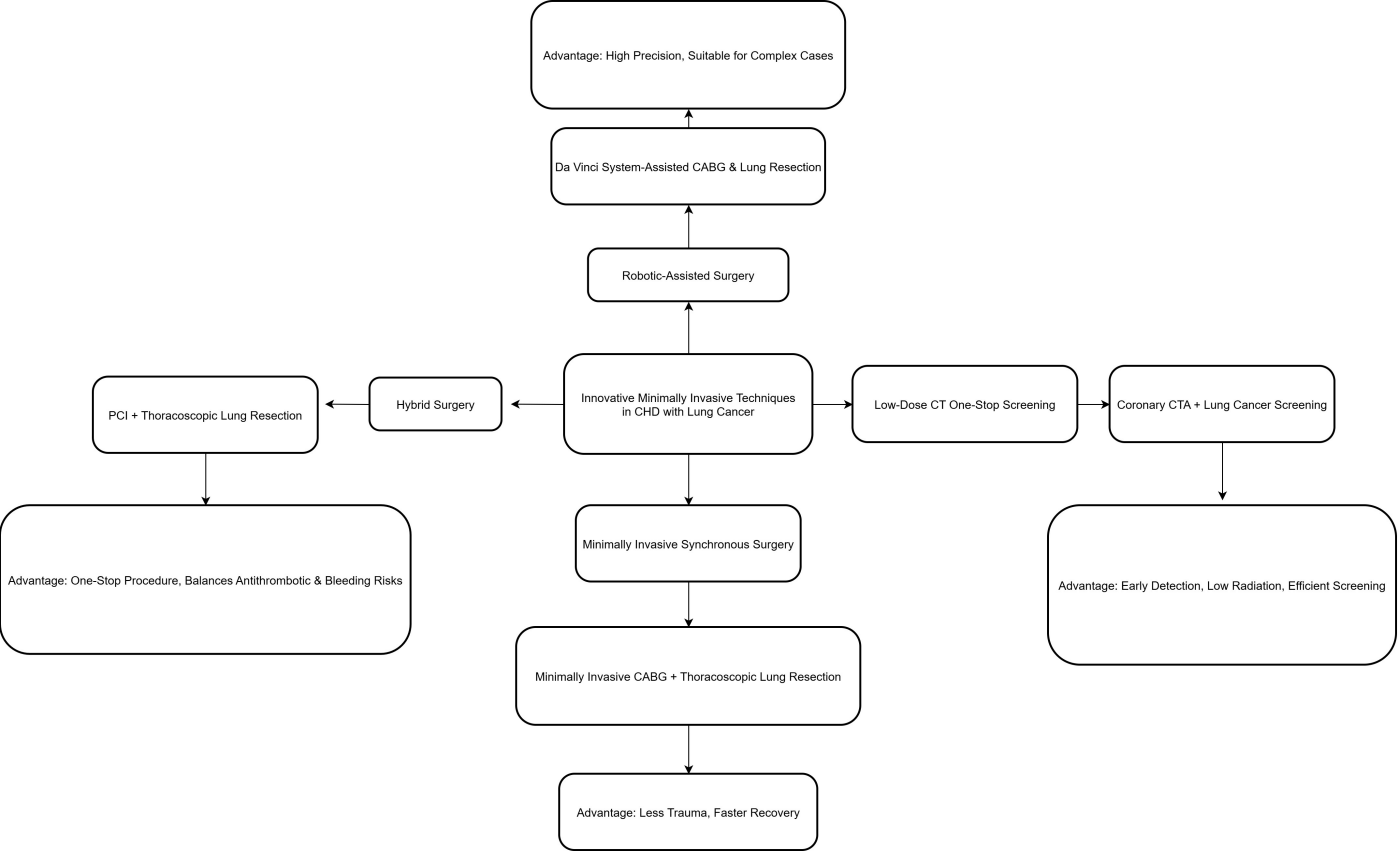
**Innovative minimally invasive techniques**. CHD, coronary heart disease; CTA, computed tomographic angiography.

The development of minimally invasive techniques has provided new strategies for surgery in CHD complicated with lung cancer, with a focus on reducing surgical trauma and shortening postoperative recovery time through technical integration, mainly through the combination of minimally invasive coronary revascularization (including PCI) and thoracoscopic lung cancer resection [12]. Zeng et al. [13] proposed an innovative hybrid procedure involving PCI and lung cancer resection. In their hybrid operating procedure, drug-eluting stents were initially implanted via PCI, followed by thoracoscopic lobectomy within 1 hour. All 14 patients achieved successful outcomes, with a 100% survival rate at 12 months after surgery. Subsequently, only two patients (14.29%) died of distant metastasis at 12–24 months, and there were no complications, such as intraoperative massive hemorrhage or in-stent thrombosis. However, it should be noted that this promising data is based on a very small initial case series (n = 14). The broader implementation of such hybrid techniques faces several challenges that warrant discussion. Firstly, the feasibility hinges on the availability of specialized hybrid operating rooms equipped for both interventional cardiology and thoracic surgery, which may limit widespread adoption. Secondly, the procedure requires a highly coordinated multidisciplinary team with expertise in both PCI and thoracoscopic surgery, implying a significant learning curve. Potential limitations include the need for precise perioperative antithrombotic management to balance stent thrombosis and surgical bleeding risks in a condensed timeline, which may not be generalizable to all centers. Furthermore, the long-term oncological outcomes and cardiovascular safety compared to established staged approaches require validation in larger, multicenter studies. The key to this technology lies in the optimization of perioperative antithrombotic regimens, which not only balance the risks of bleeding and thrombosis but also avoid the problem of tumor progression in staged surgery, providing a new treatment option for high-risk patients. Furthermore, the application of thoracoscopic technology in lung cancer resection further reduces the surgical trauma. Lim et al. [14] compared the effects of thoracoscopic and open lobectomy in staged surgery, showing that the postoperative pain score in the thoracoscopic group was significantly lower than that in the open group (visual analogue scale, 3.2 ± 1.1 vs. 5.8 ± 1.5), the postoperative hospital stay was shortened by 3.5 days, and the incidence of pulmonary infection was lower (7.1% vs. 18.6%), suggesting that minimally invasive techniques can improve the quality of postoperative recovery in patients, which is particularly suited for elderly comorbid patients with poor lung function (Fig. 2).

### 2.3 Supplementary Role of Non-Surgical Treatment

**Fig. 3.**
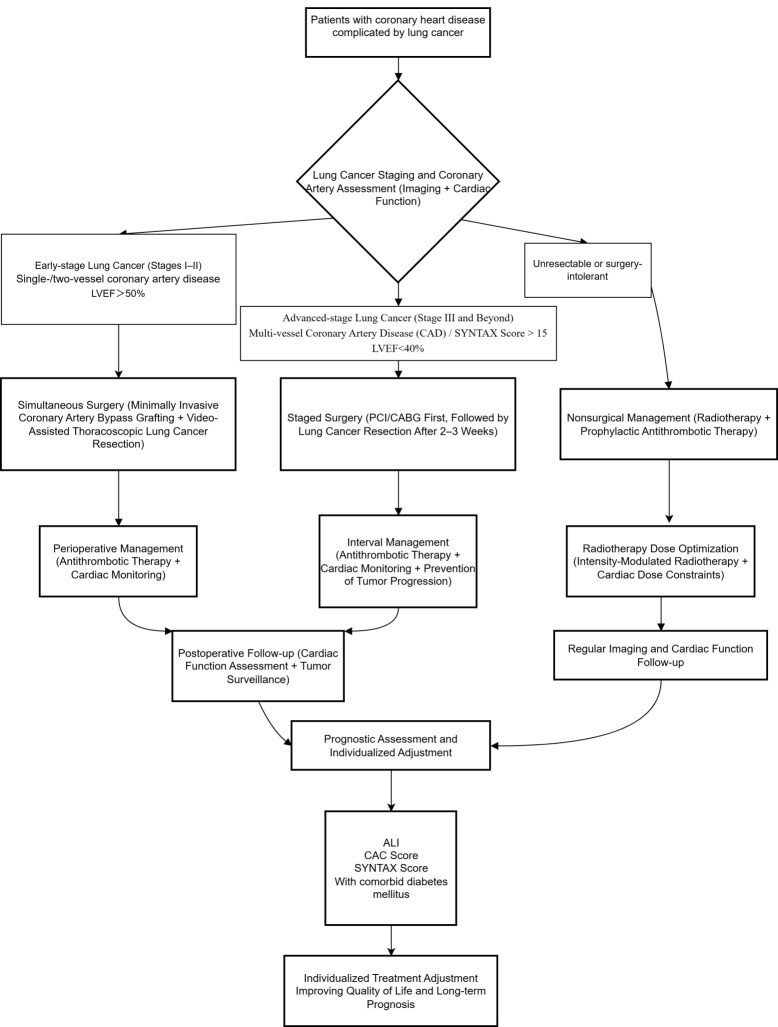
**Diagnostic and treatment flow chart**.

For patients with unresectable lung cancer (e.g., advanced NSCLC) or patients who cannot tolerate surgery, non-surgical treatment (e.g., radiotherapy, chemotherapy combined with coronary risk management) becomes the main option, but attention must be paid to the potential cardiac damage caused by radiotherapy [15]. In recent years, immunotherapy (e.g., immune checkpoint inhibitors) and targeted therapy (e.g., tyrosine kinase inhibitors) have also become important treatment modalities for advanced lung cancer. However, their potential cardiovascular toxicities, such as myocarditis, pericarditis, and hypertension, warrant careful cardiac monitoring in patients with comorbid CHD. Yakupovich et al. [16] evaluated lung cancer patients complicated with CHD who received thoracic radiation therapy, finding that the progression rate of coronary artery calcification (CAC) in patients with total cardiac radiation dose >20 Gy was significantly higher than that in patients with dose <20 Gy (42.3% vs. 18.7%), while the incidence of cardiovascular events (e.g., angina pectoris, myocardial infarction) was increased by 2.3 times within 2 years after radiotherapy. To address this, Wang et al. [17] proposed an optimized radiotherapy dose strategy, in which the average cardiac dose was controlled within 15 Gy through intensity-modulated radiation therapy, combined with preoperative CAC assessment. Using this strategy, they found that prophylactic antithrombotic therapy for patients with CAC ≥100 reduced the incidence of cardiovascular events after radiotherapy to 12.1% (Fig. 3).

## 3. Research Progress on Prognostic Influencing Factors for Coronary Heart Disease Complicated With Lung Cancer

### 3.1 Nutritional-Inflammatory Status: Advanced Lung Cancer Inflammation Index as the Core Indicator

Nutritional-inflammatory imbalance is a common pathophysiological basis for malignant tumors and cardiovascular diseases, and its impact on the prognosis of CHD patients complicated with lung cancer has become a research hotspot in recent years. The advanced lung cancer inflammation index (ALI), which integrates nutritional (body mass index, albumin) and inflammatory (neutrophil-to-lymphocyte ratio) indicators, has been proven to be an effective tool for predicting all-cause mortality in patients [18]. Lim et al. [14] conducted a Cox regression analysis based on data for 1295 CHD patients in the National Health and Nutrition Examination Survey from 1999 to 2018, finding that the all-cause mortality rate in the highest quartile group (ALI >35) was significantly lower than that in the lowest quartile group (ALI <15) (hazard ratio [HR] = 0.42, 95% confidence interval [CI] 0.28–0.63, *p* < 0.001). The findings from this large retrospective cohort are compelling, but their causal inference is limited by the observational study design. Moreover, a subgroup analysis revealed that the association was more significant in CHD patients complicated with lung cancer (HR = 0.38, 95% CI 0.21–0.69). A subsequent restricted cubic spline analysis confirmed a linear negative correlation between ALI and all-cause mortality, suggesting that ALI can serve as a simple and reliable indicator for prognostic evaluation in such patients, and providing a theoretical basis for clinical improvement of the prognosis through nutritional support (e.g., albumin supplementation) and anti-inflammatory therapy.

### 3.2 Characteristics of Coronary Artery Lesions: Predictive Value of Calcification Degree and Lesion Severity

The objective characteristics of coronary artery lesions (e.g., calcification degree, stenosis range) directly reflect the cardiac reserve function, and the impact of these characteristics on the surgical risk and long-term prognosis of CHD patients complicated with lung cancer has been verified by multiple studies.

#### 3.2.1 Prognostic Significance of Coronary Artery Calcification

CAC is a stable marker of atherosclerosis and can be quantitatively evaluated on computed tomography (CT) images (e.g., Agatston score) [19]. Atkins et al. [20] analyzed the radiotherapy planning CT images of 428 patients with locally advanced lung cancer, showing that the all-cause mortality rate (HR = 1.87, 95% CI 1.32–2.65) and the incidence rate of major adverse cardiovascular events (HR = 2.11, 95% CI 1.45–3.07) in patients with CAC ≥1 were significantly higher than those in patients with CAC = 0. This well-designed retrospective analysis provides strong associative evidence, but its prognostic utility should be further tested in prospective settings. Mendoza et al. [21] further found that incidentally detected CAC (especially Agatston score >400) on low-dose CT images for lung cancer screening increased the risk of 30-day postoperative cardiovascular complications by 3.2 times in patients, suggesting that CAC can be used as an important basis for preoperative risk stratification and that enhanced perioperative cardiac monitoring is required for patients with high CAC scores. It is worth noting that lung cancer surgery itself may cause dynamic changes in CAC. The results of this single-center retrospective study highlight an important risk factor but require external validation. Kutluk et al. [22] compared preoperative and postoperative CAC in patients who underwent pneumonectomy, finding that the 6-month postoperative CAC score was significantly higher than the preoperative score (39 ± 12 vs. 27 ± 9, *p* < 0.05). They speculated that this finding was related to the progression of atherosclerosis caused by surgical stress, indicating that regular follow-up and monitoring of CAC changes after surgery are needed to achieve timely interventions for cardiovascular risk factors.

#### 3.2.2 Impact of Coronary Artery Lesion Severity (SYNTAX Score)

The SYNTAX score is used to assess the complexity of coronary artery lesions through coronary angiography and is a classic indicator for guiding coronary revascularization strategies in CHD. Sun et al. [23] conducted a cross-sectional study involving 75 lung cancer patients complicated with CHD, finding that the incidence of postoperative complications in patients with SYNTAX score >15 was significantly higher than that in patients with SYNTAX score ≤15 (48.0% vs. 22.7%, *p* = 0.012). The cross-sectional design of this pilot study limits causal interpretation, and the small sample size warrants cautious generalization of the findings. Moreover, their multivariate logistic regression analysis identified a high SYNTAX score (odds ratio [OR] = 3.12, 95% CI 1.25–7.78) as an independent risk factor for postoperative cardiovascular complications, suggesting that preoperative assessment of coronary artery lesion severity using the SYNTAX score can assist with the screening of suitable patients for surgery and reducing perioperative risks.

### 3.3 Comorbidities and Treatment-Related Factors

In addition to the characteristics of the two main diseases, the presence of other underlying diseases (e.g., diabetes mellitus [DM]) and treatment-related measures (e.g., antithrombotic regimens) can have an important impact on the prognosis of patients. Tas et al. [24] analyzed 378 small cell lung cancer (SCLC) patients complicated with CHD, showing that the median survival time of patients with comorbid DM was significantly shorter than that of patients without DM (12.6 months vs. 18.3 months, *p* = 0.003), while hypertension and chronic obstructive pulmonary disease (COPD) had no significant impact on the survival time. These findings suggest that DM may be a negative prognostic factor for such patients by aggravating the inflammatory response and vascular damage, and that clinical strengthening of blood glucose control in patients with comorbid DM is necessary. This retrospective analysis identifies DM as a significant prognostic factor, but confirmation through prospective cohorts is needed. Among treatment-related factors, the choice of antithrombotic regimens is particularly critical. Kitamura et al. [25] conducted a multicenter study involving 1254 lung cancer patients complicated with CHD, finding that the incidence of in-stent thrombosis in patients who received standardized postoperative antiplatelet therapy (aspirin + clopidogrel dual antiplatelet therapy [DAPT]) for 6 months was significantly lower than that in patients who received a single antithrombotic drug (0.8% vs. 3.5%, *p* = 0.021). Furthermore, there was no increase in the bleeding risk (bleeding rate, 4.2% vs. 3.8%, *p* > 0.05). Thus, in the absence of contraindications, postoperative DAPT can be the preferred option to balance the risks of thrombosis and bleeding. As the largest retrospective study in this area, it provides relatively strong evidence; however, the lack of randomization means residual confounding cannot be fully excluded (Table 3).

**Table 3.** **Prognostic factors and their hazard ratios**.

Prognostic factor	Risk category	Hazard ratio (HR)/Association	Clinical implication & Recommendation
Low Advanced Lung Cancer Inflammation Index (ALI)	Nutritional-Inflammatory Status	HR = 0.42 (Highest vs. Lowest Quartile)	ALI <15 indicates poorer prognosis; recommend nutritional support & anti-inflammatory therapy
High Coronary Artery Calcification (CAC) Score	Coronary Lesion Characteristic	HR = 1.87 (CAC ≥1 vs. CAC = 0)	Higher all-cause mortality & CV event risk; strengthen cardiac monitoring
High SYNTAX Score (>15)	Severity of Coronary Lesions	OR = 3.12 (Postoperative Complication Risk)	Preoperative SYNTAX assessment recommended; high score = increased surgical risk
Comorbid Diabetes Mellitus (DM)	Comorbidity	Median survival shorter (12.6 vs. 18.3 months)	DM is a negative prognostic factor; intensive glycemic control recommended
Standardized Antiplatelet Therapy (DAPT)	Treatment-Related Factor	Lower stent thrombosis rate (0.8% vs. 3.5%)	If no contraindication, recommend 6-month DAPT

OR, odds ratio; CV, cardiovascular.

## 4. Innovative Progress in Treatment Technologies for Coronary Heart Disease Complicated With Lung Cancer

### 4.1 Simultaneous Screening Technology for Multiple Diseases: One-Stop Application of Low-Dose CT

The shared risk factors of CHD and lung cancer (e.g., smoking) make simultaneous screening possible. The development of low-dose CT technology has realized a one-stop assessment for both conditions [26,27], with the benefits of both improving the screening efficiency and reducing the radiation exposure [28]. Gaudio et al. [29] conducted a randomized controlled trial (RCT), demonstrating that simultaneous screening for CHD and lung cancer in high-risk populations using an ultrafast low-dose CT protocol (80 kVp, 15 mAs) showed no significant difference in the clear display rate of coronary segments compared with standard cardiac CT (97% vs. 99%, *p* > 0.05), and that the effective radiation dose was significantly lower than that in the separate screening protocol (3.2 ± 0.8 mSv vs. 8.5 ± 1.2 mSv). The RCT design provides a high level of evidence supporting the efficacy and safety of this protocol. Zanon et al. [30] further verified the clinical value of this simultaneous screening technology. Specifically, among 175 patients with suspected CHD, coronary CT angiography combined with additional ultra-low-dose CT lung cancer screening detected pulmonary nodules in 71 of 175 patients (41%), of which 22 cases (31%) were malignant or highly suspected malignant, suggesting that this technology can achieve early detection of lung cancer while evaluating coronary artery lesions. Therefore, this technology is particularly suitable for high-risk populations, such as smokers, and provides a new strategy for saving clinical medical resources and improving screening efficiency. This retrospective validation study supports the utility of the technology, but its real-world effectiveness would be best confirmed by prospective implementation studies.

### 4.2 Integration of Minimally Invasive Treatment Technologies: Hybrid Surgery and Robot-Assisted Surgery

The integrated innovation of minimally invasive technologies has further expanded the treatment boundaries for CHD complicated with lung cancer, mainly through the application of hybrid surgery (which often incorporates PCI as a component) and robot-assisted surgery. Zeng et al. [13] proposed PCI-lung cancer resection hybrid surgery, which integrates vascular intervention and thoracoscopic technology in a hybrid operating protocol. All 14 patients completed the connection between PCI and lobectomy within 1 hour, with a 100% survival rate at 12 months after surgery, thus solving the contradiction between antithrombotic therapy and surgical bleeding in traditional staged surgery. As previously mentioned, while these initial results are encouraging, the small sample size necessitates cautious interpretation. The feasibility on a larger scale depends on addressing infrastructural requirements (hybrid ORs), developing standardized protocols, and training specialized teams, which represent significant initial investments. Yeginsu et al. [31] explored robot-assisted synchronous surgery, completing minimally invasive CABG combined with lung cancer resection using the da Vinci robotic system. They reported that 10 patients had no serious complications after surgery, and that the surgical accuracy and operational flexibility were significantly better than those for traditional minimally invasive surgery, thus providing a new option for the treatment of complex cases (e.g., multivessel coronary artery lesions complicated with lung cancer). Similarly, this report involves a very limited number of patients (n = 10). Robot-assisted surgery introduces additional considerations, including high costs for the robotic system and consumables, prolonged operative times during the surgeon’s learning phase, and the need for advanced technical skills. Its potential benefits in reducing trauma and enhancing precision in complex comorbid cases must be weighed against these economic and practical constraints. More extensive clinical experience and cost-effectiveness analyses are required to define its role in this specific patient population.

## 5. Research Controversies and Future Directions

### 5.1 Core Controversies in Current Research

Ambiguous applicable boundaries for synchronous surgery: Most existing studies on synchronous surgery are single-center, small-sample retrospective analyses, and there is a lack of verification in multicenter RCTs. There are no unified standards for the applicable populations (e.g., feasibility in Stage III lung cancer patients) and surgical techniques (e.g., incision selection, anastomosis sequence), leading to blindness for the appropriate clinical application of synchronous surgery.

Insufficient clinical translation of prognostic evaluation indicators: Although ALI, CAC, SYNTAX score, and other factors have been shown to be associated with prognosis, research is still needed on how to integrate these factors into a clinical risk scoring system for rapid preoperative stratification. Moreover, the applicability differences of these indicators for different pathological types of lung cancer (e.g., NSCLC vs. SCLC) have not been clarified, thus limiting their clinical promotion.

Obvious individual differences in antithrombotic regimens: The postoperative antithrombotic course (e.g., DAPT for 3 months vs. 6 months) and drug selection (e.g., applicability of novel oral anticoagulants) need to be comprehensively judged based on the stent type, lung cancer stage, and bleeding risk in patients. However, existing research lacks recommendations for targeted individualized regimens, and clinical decisions rely on the experience of doctors, making them prone to treatment differences.

### 5.2 Future Research Directions

To focus on the treatment of CHD complicated with lung cancer, multicenter RCTs need to be conducted to clarify the cost-to-benefit ratios of different surgical and non-surgical (including PCI-based ) regimens, construct a special prognostic prediction model based on multi-omics, explore perioperative novel antithrombotic drugs and optimal medical therapy (e.g., role of GLP-1 RAs), and utilize AI technology to optimize treatment, and strengthen long-term follow-up to improve the survival quantity and quality of life for patients.

## 6. Conclusions

This review has combed the literature on the treatment of CHD complicated with lung cancer from 2016 to 2025, finding that: synchronous surgery is suitable for patients with Stage I–II lung cancer and single/double-vessel coronary artery lesions, while staged surgery or a PCI-first strategy is suitable for those with severe multivessel coronary artery lesions or advanced lung cancer; minimally invasive techniques including PCI and hybrid approaches can reduce trauma and shorten recovery time; low ALI, high CAC, high SYNTAX score, and comorbid DM are independent risk factors for poor prognosis; and simultaneous screening protocols with low-dose CT and other technologies can provide new diagnosis and treatment schemes. However, a significant portion of the existing research consists of single-center retrospective studies with small sample sizes, which constitutes a low level of evidence and limits the generalizability of the conclusions and the precision of the proposed strategies. Therefore, in the future, individualized diagnosis and treatment protocols that encompass the full spectrum of revascularization options (PCI, CABG, hybrid) and integrate modern cardioprotective pharmacotherapy need to be promoted through multicenter RCTs and other high-level evidence studies to improve the survival outcomes of patients with comorbid CHD and lung cancer.
